# Using the Information Provided by Forbidden Ordinal Patterns in Permutation Entropy to Reinforce Time Series Discrimination Capabilities

**DOI:** 10.3390/e22050494

**Published:** 2020-04-25

**Authors:** David Cuesta-Frau

**Affiliations:** Technological Institute of Informatics, Universitat Politècnica de València, 03801 Alcoi Campus, Spain; dcuesta@disca.upv.es; Tel.: +34-966528505

**Keywords:** permutation entropy, ordinal patterns, forbidden patterns, signal classification

## Abstract

Despite its widely tested and proven usefulness, there is still room for improvement in the basic permutation entropy (PE) algorithm, as several subsequent studies have demonstrated in recent years. Some of these new methods try to address the well-known PE weaknesses, such as its focus only on ordinal and not on amplitude information, and the possible detrimental impact of equal values found in subsequences. Other new methods address less specific weaknesses, such as the PE results’ dependence on input parameter values, a common problem found in many entropy calculation methods. The lack of discriminating power among classes in some cases is also a generic problem when entropy measures are used for data series classification. This last problem is the one specifically addressed in the present study. Toward that purpose, the classification performance of the standard PE method was first assessed by conducting several time series classification tests over a varied and diverse set of data. Then, this performance was reassessed using a new Shannon Entropy normalisation scheme proposed in this paper: divide the relative frequencies in PE by the number of different ordinal patterns actually found in the time series, instead of by the theoretically expected number. According to the classification accuracy obtained, this last approach exhibited a higher class discriminating power. It was capable of finding significant differences in six out of seven experimental datasets—whereas the standard PE method only did in four—and it also had better classification accuracy. It can be concluded that using the additional information provided by the number of forbidden/found patterns, it is possible to achieve a higher discriminating power than using the classical PE normalisation method. The resulting algorithm is also very similar to that of PE and very easy to implement.

## 1. Introduction

Despite its relatively young age in comparison with other entropy statistics, permutation entropy (PE) has already become one of the most utilised time series entropy-related measures. It was proposed in the well known paper by Bandt and Pompe [1] in 2002, and since then, it has given rise to a number of applications and further algorithm developments. This number is growing exponentially [2], which confirms the goodness of the PE approach.

Regarding PE applications, this measure has been used in many fields, such as medicine, engineering, seismology, and economics. In medicine, it has been frequently used as a diagnostic aid to disclose information hidden in physiological time series. The most common physiological time series processed using PE are probably electroencephalograms (EEG) and electrocardiograms, but in the form of R-wave interval series (RR records). For example, in [3], PE was applied to EEG records to find seizure-free, pre-seizure, and seizure phases. This a recurrent application in many similar studies. The work described in [4] assessed two classifiers based on logistic regression and quadratic discriminant analysis to detect obstructive sleep apnea using PE as one of the features extracted from RR records. Other biomedical signals successfully processed using PE are body temperature time series [5] and glucose records [6]. These are just a few examples of the huge number of medical applications based on PE. A more detailed survey is described in [2].

Engineering is another field that has exploited the PE capabilities. It has been mainly used in mechanical engineering for fault detection. That is the case in [7], where the authors described a mathematical tool based on PE for bearings fault type diagnosis. Many similar applications can be found in the scientific literature [7,8]. There are other novel and interesting implementations in mechanical engineering, such as [9], where PE was used to assess road flatness.

Seismology and economics are emergent fields of PE utilisation. The work in [10] used noise randomness assessed by PE to quantify its changes as a possible precursor of volcanic activity. An example of PE used in economics is [11]. This work described a method based on PE to detect changes in stock market data.

The basic PE algorithm has also been improved or customised since its initial version. PE is based on the relative frequency of ordinal patterns that can be drawn from a time series, but it overlooks the information embedded in the amplitude. As a consequence, some PE-derived methods have been devised to address this problem, such as the weighted permutation entropy [12] (WPE), the fine grained permutation entropy [13] (FGPE), and the amplitude aware permutation entropy [14] (AAPE). A comparative analysis of these measures has been recently reported in [15]. Other problems, such as order ambiguities due to equal value samples in subsequences (ties), have been addressed in [14,16], with practical analyses described in [17,18]. Other PE algorithm tweaks can be found in the scientific literature; for instance, the bubble entropy (BE) method [19], aimed at improving the robustness of PE in terms of input parameters.

However, there is another topic related to PE that has not been very much exploited in practical term—forbidden patterns. When analysing the order (either ascending or descending) of a numeric subsequence of length *m*, theoretically, any of the m! possible permutation patterns could be found. This is true for random sequences where the probability of occurrence of each ordinal pattern is not zero, provided the total length suffices to allocate even the least probable pattern. Nevertheless, if the sequence exhibits a certain degree of determinism, some order relations will never occur—the so-called forbidden patterns [20]. Actually, the numbers of admissible/forbidden patterns have been demonstrated to provide information with system distinguishing power [21], and a few studies have exploited this new feature independently of PE for signal classification [22]. Based on all these previous studies, we hypothesised that a new scheme that could combine standard PE and a metric related to the number of forbidden patterns, could better portray the dynamics of the underlying time series, and therefore result in a more powerful measure for signal classification.

Thus, this paper describes a modified PE method by means of integrating the number of ordinal patterns found (the complementary of the number of forbidden/missing patterns) in the normalisation step. With this simple customisation, the resulting new method, referred to in this paper as PE2, in contrast to the standard method, PE1, is clearly more robust and more powerful in terms of signal classification than the PE1 method, as the experiment results, using a varied and diverse set of time series, demonstrate in Section 3.

The structure of the paper is as follows. Section 2.1 describes the PE1 algorithm, followed by Section 2.2, describing the new variant proposed, PE2. Section 2.3 details the time series datasets used in the experiments. The metrics employed to quantify and assess the performance of the approaches are outlined in Section 2.4. The quantitative results of all the cases analysed are presented in Section 3. The Discussion section (Section 4) provides an interpretation of the results. Finally, the conclusions are described in Section 5.

## 2. Methods

### 2.1. Permutation Entropy

The present study is based on the original PE algorithm described in [1]. This method computes a normalised histogram of ordinal patterns found in the subsequences drawn from a time series, when sorted in ascending order, from which the Shannon entropy is calculated. The length of these subsequences is defined by an input parameter, the embedded dimension *m*.

Formally, the input time series under analysis is defined as a vector of *N* components $x=\left\{x_{0},x_{1},\ldots ,x_{N-1}\right\}$. A generic subsequence extracted commencing at sample $x_{j}$ of x is defined as a vector of *m* components $x_{j}^{m}=\left\{x_{j},x_{j+1},\ldots ,x_{j+m-1}\right\}$. In its original state, the samples in $x_{j}^{m}$ can be assigned a default growing set of indices given by $\pi^{m}=\left\{0,1,\ldots ,m-1\right\}$. The subsequence $x_{j}^{m}$ undergoes, then, an ascending sorting process, and the sample order changes in it are mirrored in the vector of indices $\pi^{m}$. The resulting new version of this vector, $\pi_{j}^{m}=\left\{\pi_{0},\pi_{1},\ldots ,\pi_{m-1}\right\}$, with $x_{j+\pi_{0}}\leq x_{j+\pi_{1}}\leq x_{j+\pi_{2}}\ldots \leq x_{j+\pi_{m-1}}$, is compared, in principle, with all the possible m! ordinal patterns of length *m*. When a coincidence is found, a specific associated counter to that pattern, $c_{i}\in c$, is increased. This process is repeated with all the possible N−(m−1) subsequences (0≤j<N−m+1) until the complete histogram is obtained. Each bin of the histogram is finally normalised by N−(m−1) in order to get an estimation of the probability of each ordinal pattern: $p=\left\{p_{0},p_{1},\ldots ,p_{m!-1}\right\}\left|p_{i}=\frac{c_{i}}{N-(m-1)}\right.$. This vector of probabilities is used to calculate PE as:

(1) $$PE\left(x,m,N\right)=-\sum_{k=0}^{m!-1}p_{k}logp_{k},\forall p_{k}>0$$

There is another input parameter for PE, the embedded delay τ. This parameter, when τ>1, defines the time scale at which PE is computed, and it can contribute to gain a deeper insight into the temporal correlations of the time series [23]. However, since this parameter is almost equivalent to a downsampling process [14], and given that the present study is a comparative study in relative terms, we took τ=1 in all the experiments, as in many other works [1,14,19,22].

Some numerical examples of PE computation can be found in the literature. For examples, see [15,18,24].

For comparative purposes, another improved version of PE will be included in some experiments, WPE [12]. The difference is to use a weight factor applied to the relative PE frequencies that quantifies amplitude values. The weighting factor for each relative frequency is given by $w_{j}=\frac{1}{m}\sum_{k=0}^{m-1}{\left(x_{j+k}-{\bar{X}}_{j}^{\;m}\right)}^{2}$, ${\bar{X}}_{j}^{\;m}$ being the arithmetic mean of $x_{j}^{m}$. The new value *W* becomes the new denominator instead of N−m−1, with $W=\sum_{j=0}^{N-m}w_{j}$. Further WPE details are described in [15].

### 2.2. Permutation Entropy Using the Number of Patterns Found

Forbidden patterns, in the sense of patterns that will never occur in a sequence regardless of its length, have been demonstrated to provide additional information about the determinism degree of the underlying time series [21]. These forbidden patterns can even be considered as a new dynamical property [21,25], and have already been used successfully as a quantifier to assess stock market inefficiency [22]. In cases of unobserved patterns due to low probabilities of occurrence in a relatively short time series, namely, not truly forbidden but missing patterns [25], they can also be considered potential distinguishing features if all the records are balanced with regard to length [26]. The numbers of forbidden and admissible patterns are two sides of the same coin, since they are complementary and totalise the theoretical number of possible patterns m!.

In a more formal way, if the probability of an ordinal pattern $\pi_{i}$ is $P_{\pi_{i}}$, for an admissible pattern $P_{\pi_{i}}>0$, whereas $P_{\pi_{i}}=0$ for a forbidden pattern (these probabilities can be thought of as relative frequencies for finite length time series). A forbidden pattern implies there is no $x_{j}^{m}$ in x for which the ordinal pattern is $\pi_{i}$. The presence of a forbidden pattern is heuristically linked to determinism [25], and its presence causes an even higher number of forbidden patterns for longer subsequences, which can also be exploited from a classification point of view, as will be described in the experiments later. For example, if $\pi_{i}^{3}=\left\{2,1,0\right\}$ is a forbidden pattern of x, for m=4, $\left\{3,2,1,0\right\}$, $\left\{2,3,1,0\right\}$, $\left\{2,1,3,0\right\}$, and $\left\{2,1,0,3\right\}$ will be also forbidden patterns [20], and so on.

Periodic sequences excellently illustrate the concept of forbidden patterns (a purely deterministic time series). Let $x=\left\{1\right.$, 2, 3, 1, 2, 3, 1, 2, 3, 1, 2, 3, …, 1, 2, $\left.3\right\}$ be a periodic sequence of length *N* and period 3. All the subsequences of length m=3 that can be extracted from x are $x_{0}^{3}=\left\{1,2,3\right\}$ , $x_{1}^{3}=\left\{2,3,1\right\}$ , $x_{2}^{3}=\left\{3,1,2\right\}$ , $x_{3}^{3}=\left\{1,2,3\right\}$ , $x_{4}^{3}=\left\{2,3,1\right\}$ , $x_{5}^{3}=\left\{3,1,2\right\}$ , $x_{6}^{3}=\left\{1,2,3\right\}$ , $x_{7}^{3}=\left\{2,3,1\right\}$ , $x_{8}^{3}=\left\{3,1,2\right\}$ , $x_{9}^{3}=\left\{1,2,3\right\}$ , $x_{10}^{3}=\left\{2,3,1\right\}$ , $x_{11}^{3}=\left\{3,1,2\right\}$ , …, $x_{N-m}^{3}=\left\{1,2,3\right\}$.

When the previous subsequences are ordered, the results are: $\pi_{0}^{3}=\left\{0,1,2\right\}$ , $\pi_{1}^{3}=\left\{2,0,1\right\}$ , $\pi_{2}^{3}=\left\{1,2,0\right\}$, and these three motifs keep repeating indefinitely. It can be easily concluded that motifs $\left\{0,2,1\right\}$, $\left\{1,0,2\right\}$, and $\left\{2,1,0\right\}$, will never be found in x, even for N→∞. These three motifs could therefore be considered forbidden patterns.

Given that PE looks at the dynamics of a time series in terms of relative frequency of ordinal patters, but overlooks the additional information provided by the number of forbidden/admissible patterns (which is also related to the randomness of the time series [20]), we hypothesised that there could exist a potential synergy between the two sources. After studying several integration possibilities, a straightforward and simple solution was to change the PE probability normalisation factor N−m+1, which accounts for the number of subsequences that can be drawn from the time series, by the actual number of different ordinal patterns found, termed *T*.

In principle, PE becomes non-normalised this way, since $\sum_{\forall p}p_{k}\neq 1$ in most cases. The *T* value can be considered a rescaling or weighting factor that embeds the forbidden patterns information in the modified PE measure, PE2, and its additional class discriminating power would be lost if an individual normalisation took place on a signal by signal basis (intrinsic). In order to keep the PE2 results within a reasonable interval, as with other similar measures, including PE, it is recommended to adopt a global feature normalisation scheme (extrinsic) after PE2 computation, if normalisation is desired.

There are many feature normalisation methods reported in the scientific literature: Z-Score, Min-Max, and other linear and non-linear scaling methods [27]. In case it is necessary, we propose using a linear proportionate normalisation scheme [28]. Each PE2 value can be divided by the sum of all the PE2 values. Therefore, each result accounts for its relative contribution within the entire set of results; it does not destroy proportionality (namely, discriminating power), and the newly computed value can be easily related to the original value. In other words, order and differences are not lost or modified. Moreover, it is not based on arbitrary choices, and its implementation is straightforward. An even more convenient variation of this method would be to divide the PE2 values by the maximum PE2 value obtained in order to keep the range of possible results between 0 and 1 [29], more easily interpretable.

For example, in [15] the sequence $x=\left\{-0.45\right.$, 1.9, 0.87, −0.91, 2.3, 1.1, 0.75, 1.3, −1.6, 0.47, −0.15, 0.65, 0.55, −1.1, $\left.0.3\right\}$ was analysed from a PE perspective (N=15,m=3). As a result, the counter of the frequencies for each ordinal pattern obtained was $c=\left\{0,4,3,1,2,3\right\}$, with $p=\left\{0,0.31,0.23,0.08,0.15,0.23\right\}$, using N−m+1=13 as the normalisation factor. Since the ordinal pattern $\left\{0,1,2\right\}$ was not found (a missing pattern, because x is a random sequence and $\left\{0,1,2\right\}$ would occur if given more samples), applying PE2, p would have instead given $p=\left\{0,0.8,0.6,0.2,0.4,0.6\right\}\left(T=5\right)$, from which PE2$\left(x,3,15\right)=2.135$ whereas PE1 was PE1$\left(x,3,15\right)=2.20$ [15]. Once the PE2 values from all the time series under analysis were computed, the normalisation scheme described above could be applied, although it would not be necessary for classification purposes because the differences would already be in the resulting PE2 values.

As for the PE1 algorithm, a PE2 algorithm can be implemented in many ways, but we have chosen to use a bubble sort approach to obtain the ordered indices, and dynamically update the list of different ordinal patterns found, termed $\Pi^{m}$ (initially empty), instead of assuming a set with all the possible m! permutations. This implementation is less computationally efficient due to the list operations (searching and appending), but it is better suited to implementing the improvements devised in the PE2 algorithm. Besides, it can be more memory-efficient, since, in case of forbidden patterns, as in many chaotic time series [22,30,31], there is no need to store all the theoretically possible m! ordinal patterns, only those really found in the data. This could entail significant memory savings when *m* is relatively large and/or forbidden patterns are frequent [26]. Last but not least, a linked-list facilitates the implementation and even integration of other PE variants based on a dynamic generation of patterns, such as FGPE [13]. Based on this approach, a suggested implementation is shown in Algorithm 1.
**Algorithm 1** Permutation entropy scaled by the number of patters found (PE2) algorithm. **Input:**
x, m>2, N>m+1 **Initialisation:** PE2 ←0, $c\leftarrow \left\{⌀\right\}$, $p\leftarrow \left\{⌀\right\}$, $\Pi^{m}\leftarrow \left\{⌀\right\}$ **for**
j←0,…,N−m
**do**   $x_{j}^{m}\leftarrow \left\{x_{j},x_{j+1},\ldots ,x_{j+m-1}\right\}$     $\pi^{m}\leftarrow \left\{0,1,\ldots ,m-1\right\}$   bSorted ← false     **while** (bSorted=false) **do**▹ Bubble sort    bSorted ← true      **for**
i←j,…,j+m−2
**do**      **if**
$\left(x_{i}>x_{i+1}\right)$
**then**         swap$\left(x_{i},x_{i+1}\right)$         swap$\left(\pi_{i},\pi_{i+1}\right)$         bSorted ← false        **end if**      **end for**    **end while**    bFound ← **false**     **for**
$i\leftarrow 0,\ldots ,\mathrm{sizeof}\left(\Pi^{m}\right)$
**do**▹ List search     **if**
$\pi_{j}^{m}=\Pi_{i}^{m}$
**then**▹ Ordinal pattern found      $c_{i}\leftarrow c_{i}+1$▹ Update counter      bFound ← **true**        **break**▹ Pattern found, exit loop     **end if**   **end for**    **if not** bFound **then**▹ Ordinal pattern not found     $\Pi^{m}⇐\pi_{j}^{m}$▹ Append pattern to list     c⇐1▹ Append and initialise pattern count   **end if**  **end for**  $T=\mathrm{sizeof}\left(\Pi^{m}\right)$ **for**
i←0,…,T
**do**    $p⇐p_{i}\leftarrow \frac{c_{i}}{T}$▹ DESNORMALISATION    PE2 ← PE2+$\left(-p_{i}\log p_{i}\right)$ **end for** **Output:** PE2$\left(x,m,N\right)$

The PE2 algorithm can become equivalent to PE just by replacing *T* by N−m+1 at the line labelled DESNORMALISATIONmathsizesmall in Algorithm 1. If the records are very short, it is not just a question about forbidden patterns but about the low probabilities of certain ordinal patterns. If all the classes exhibit the same behaviour in terms of length, this should not be an influencing factor; otherwise, a length normalisation scheme should be devised.

### 2.3. Experimental Dataset

First, the addition of synthetic databases was considered, since this kind of records is also very useful for characterising the performances of methods under more controlled conditions. In a very recent paper [32], we proposed to use a hidden Markov model to create synthetic records based on transition probabilities of their ordinal patterns of length m=3. This is a very suitable tool to create a synthetic dataset for the present study, since the main difference between PE1 and PE2 is the use of the number of actual ordinal patterns found. Assigning a 0 probability to some ordinal pattern transitions, the frequency of the destination pattern can be minimised, and for m>3, the probability of derived patterns is likely to reach 0, since the number of forbidden patterns grows superexponentially [20].

**Figure 1:**
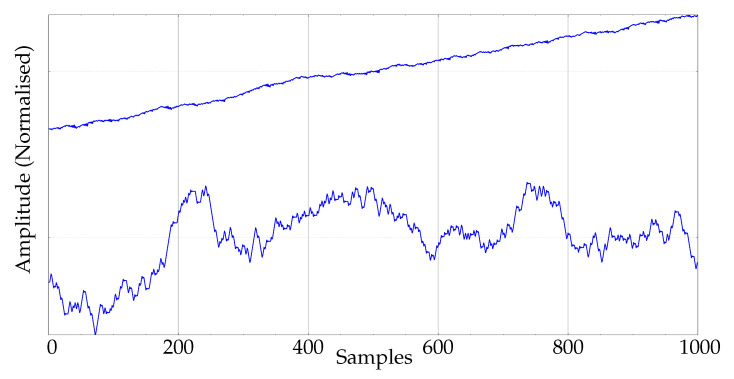
Example of synthetic signals of the experimental database.

$a_{ij}$ being the transition probability between consecutive states $q_{i}$ and $q_{j}$ at time *t*, $a_{ij}=p\left[State_{t+1}=q_{j}|State_{t}=q_{i}\right]$, and the following correspondence between model states and ordinal patterns: $q_{0}\leftarrow \left(0,1,2\right)$, $q_{1}\leftarrow \left(0,2,1\right)$, $q_{2}\leftarrow \left(1,0,2\right)$, $q_{3}\leftarrow \left(1,2,0\right)$, $q_{4}\leftarrow \left(2,1,0\right)$, and $q_{5}\leftarrow \left(2,0,1\right)$, 100 records of two synthetic classes were generated using this model. For one class, the transition probabilities were $a_{ij}=\left\{0.5,0.5,0.0\right\}$, and for the second class $a_{ij}=\left\{0.5,0.0,0.5\right\}$, probabilities defined as in [32]. Therefore, each class penalised a different transition, and that would impact the number of patterns found at m=4 and beyond in a different way for each class, since the model is not symmetric (see details in [32]). An example of the resulting signals is shown in Figure 1. The experiments on these records used 10 random realisations in each test.

**Figure 2:**
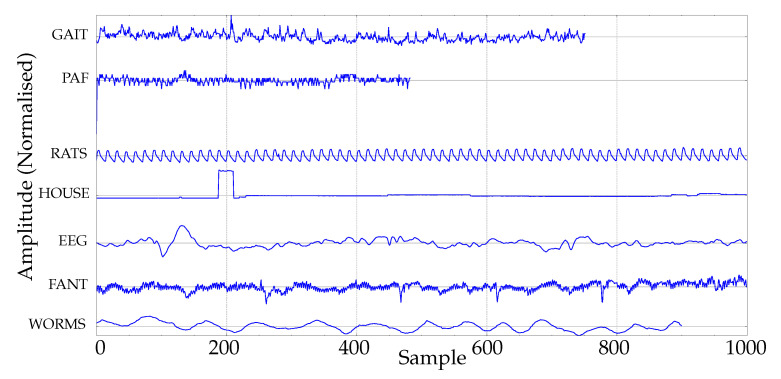
Example of real signals of the experimental database.

In addition, a real experimental dataset was chosen with the primary goal of assembling a publicly available collection of widely representative data of the most common time series entropy applications. This would enable other researchers to replicate the experiments and draw sound conclusions about the most likely performance under a disparity of conditions, not just for a single dataset/case, as occurs in many studies. Figure 2 depicts an example of one signal from each dataset used, described next:

- BONN. This database was collected at the Department of Epileptology, University of Bonn [33], and is a frequently used dataset found in many similar research studies [17,34,35,36,37,38]. The length of the records is 4097, with two classes of 100 time series each, corresponding to seizure-free and seizure-included electroencephalograms (EEGs). This dataset was chosen due to its popularity among the scientific community, and because EEGs are the focus of many entropy-related studies.
- GAIT. The records included in this dataset were drawn from Physionet gait in the ageing and disease database [39]. Although this is a small collection of gait data, with only five subjects per class, we found it very representative of another group of physiological data, not as common as EEGs, and useful to explore algorithms’ performances. The 15 records correspond to five healthy young adults, five healthy old adults, and five old adults with Parkinson’s disease. The data are stride intervals [40]. The length of the records is around 800 samples for healthy subjects, and it is 200 for pathological ones, which suffices for a representative classification analysis using PE, according to recent studies [26]. Anyway, a variation of this subset, termed GAIT2, where all the records were cut short to 200 samples, was included in the experiments for comparative purposes.
- FANT. The fantasia dataset contains 120 min of electrocardiographic and respiration data from 20 young and 20 elderly healthy subjects, and it is also available at [39]. Only the RR-interval time series were used in the experiments in this paper. RR records are also a field of intensive research [4,41,42,43,44]. A detailed description of this database can be found in [43].
- RATS. Records of blood pressure readings from Dahl SS rats on high and low salt diets [45]. The database contains nine records of each class, sampled at 100 Hz, with a total length of 12,000 samples [39].
- WORMS. This database corresponds to the recorded 2D movement of genetically-modified worms [46,47,48], and is publicly available at www.timeseriesclassification.com. It was included to have a dataset not related to physiological records, and to widen the scope of the analysis. Specifically, there were 181 records of two classes (76 wild-type and 105 mutant type) of length 900 in this subset used in the experiments.
- HOUSE. The records in this database are also publicly available at www.timeseriesclassification.com, and they correspond to non-physiological data too. There are two classes of 20 records each, with 1022 samples per record [49].
- PAF. This dataset contains paroxysmal atrial fibrillation (PAF) records [50]. There are two classes of 25 records, each one (PAF and PAF-free episodes) five minutes in duration. These records were also drawn from [39].

### 2.4. Performance Analysis

The performance of each approach under analysis was quantified using the classification accuracy: ratio of correctly classified records. Significance of this classification was qualitatively assessed by means of sensitivity (Se) and specificity (Sp), since very unbalanced results (for example, 0.7 accuracy with Se = 0.4 and Sp = 1 is not considered significant, at least a minimum 0.6 result is required), reflect an underlying poor discriminating power, regardless of the global classification accuracy. The classification threshold was taken as the ROC (receiver operating characteristic) curve point closest to the (0,1) coordinates [51]. It is important to note that the goal of the study was not to design an optimal classifier, but to carry out a fair comparison between the performances of the two measures tested under the same conditions.

The quantitative significance of the classification accuracy was assessed by means of an unpaired Wilcoxon–Mann–Whitney test. This is a very robust test that does not require data normality [52]. The significance threshold was set at α=0.05.

## 3. Results

The experiments were first carried out using the synthetic dataset described before. Since the transition probabilities were quite different, both PE1 and PE2 were capable of finding significant differences between the two classes generated. These results are shown in Table 1. It is important to note that as *m* increased, and the differences between classes in terms of patterns found became greater, and so did the PE2 accuracy, whereas PE1 performance was lower.

Table 2 shows the classification performances and statistical significance of the results achieved by the standard PE1 on real records. These experiments also included a variation of the parameter *m*, from 3 to 8, since input parameter influence is another topic of intense debate and research in the scientific literature.

The previous experiments for PE were repeated using the new approach: PE2. These additional results are shown in Table 3.

In order to better support the addition of the number of admissible/forbidden patterns in the PE method, this number was computed for each dataset. The results of this experiment are shown in Table 4. A great difference between the number of patterns found for the classes under comparison, would suggest that the addition of this number could make a significant contribution to the discriminating power of PE.

**Figure 3:**
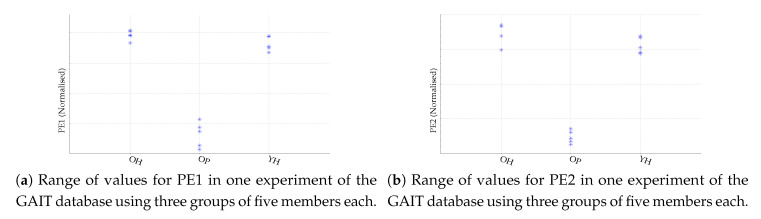
Range of values for PE1 and PE2 using the optimal *m* value to achieve the maximum possible classification accuracy in each case, using the three classes in the GAIT database (OH: old, healthy; OP: old, Parkinson’s; YH: young, healthy). The OP class is clearly distinguished from the other two in both cases. The other two groups overlap in one or two class members.

**Figure 4:**
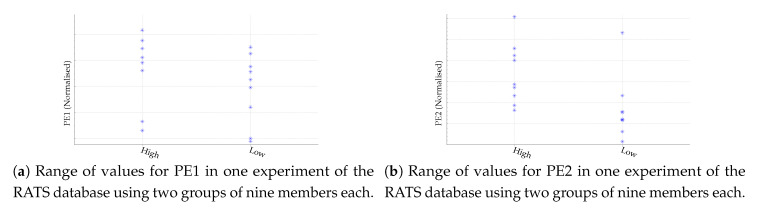
Range of values for PE1 and PE2 using the optimal *m* value to achieve the maximum possible classification accuracy using the two classes in the RATS database (High: high salt diet; Low: low salt diet). There is an overlapping of three or four members in PE1 for both classes, and that is surely why PE1 did not achieve statistical significance. For PE2, all the members in class High can be classified correctly, with an overlapping of the two top members of the Low class. This corresponds to the numerical results 0.77, 1, and 0.88 in Table 3.

Despite the results shown in Table 2 and Table 3, some of them could be debatable due to the low number of subjects, especially in the case of the GAIT database. For this reason, we have included two plots of the PE1 and PE2 values obtained to visually check the validity of the classification results for the GAIT database and for the RATS database, with nine members in each class. These plots are depicted in Figure 3 and Figure 4 respectively. Anyway, it is important to note that the analysis should be viewed in comparative terms. We are not proposing a classifier for this GAIT dataset; we are assessing whether PE2 performs better than PE1 or not.

A summary of the best statistically significant performance achieved using the two approaches assessed (PE1,PE2) is shown in Table 5 for real records. As hypothesised, the combination of PE and the actual number of patterns, the newly proposed scheme PE2, achieved the highest accuracy, and was more robust (only one dataset was not significant), than PE alone. Since in a previous study [15], WPE exhibited the best performance of a group of PE algorithm improvements, Table 5 also includes the results of applying this method, along with a denormalised version (WPE + number of patterns found) using the same approach as for PE2, since WPE also enables the computation of the measure without including the number of expected patterns. WPE has also outperformed PE in other studies, such as in [53,54].

Other factors that are taken into account to assess the utility of an entropy measure are its dependence on parameters or artefacts, such as time series length and robustness against noise. The results of a length influence analysis are shown in Table 6. In this case, PE1 and PE2 were compared using *m* values for which their performances were most similar and significant, and datasets with at least 750 samples in their time series.

The results of the noise robustness test are shown in Table 7. It is important to note that the time series were probably already affected by noise, whose level was not known. Therefore, this test considered the signals completely free of noise when computing the resulting SNR, but in reality it should be considered lower in practical terms. The levels tested were 40, 30, 25, 20, and 15 dB SNR. The noise was a random uniform time series added to the experimental datasets, with 10 realisations for each test.

## 4. Discussion

There was a clear correlation in Table 1 between differences in patterns found and accuracy achieved using PE2 in synthetic records. Since the ordinal patterns were generated using a length of 3, for m=3, and its multiple m=6, the performance of PE1 was high, but far lower for other *m* values used in the computation. Those patterns scarcely or did not at all, at m=3, give rise to a set of forbidden patterns for m>3. The number of forbidden patterns grows superexponentially [20], and that is why the differences between classes became more apparent for higher *m* values, and only PE2 was able to take advantage of this effect.

PE2 yielded a higher classification accuracy than PE1 for all the experimental real datasets except PAF, and it was equal in the GAIT case (Table 5). Moreover, PE1 was unable to find differences in three out of the seven datasets, whereas PE2 only failed in one. Therefore, it can arguably be concluded that PE2 has a greater discriminating power than PE1. The experimental dataset was reasonably very varied and diverse, chosen from publicly available repositories, and used in other similar works.

It is important to note that the PE1 and PE2 results for low *m* values were very similar. This is probably due to the fact that there is no significant difference in terms of number of patterns found between classes for such low values. In fact, these differences become more apparent beyond m=5 (Table 4).

Specifically, results for the BONN database were Se = 0.93, Sp = 0.90, and Acc = 0.91 for PE with m=3, and 0.90, 0.97, and 0.93 respectively, for PE2 with m=5. This is not a great difference, probably because the PE1 performance was already very good, despite the clear differences in terms of patterns found. The situation was the same for the GAIT dataset results. For the FANT dataset, PE1 did not find significant differences for all the *m* values tested, but PE2 did for m=6,7, precisely where the relative differences between the two classes in terms of patterns found was the highest. This was also the case for the RATS dataset: PE1 failed, but PE2 found differences, with higher accuracy correlated with higher relative differences in the number of patterns found; the maximum performance being reached at m=8. The separability of the classes in the WORMS dataset was slightly improved with PE2. This could be due to the high variability in the number of patterns found, quantified in terms of standard deviation (Table 4). The records in the HOUSE dataset were not distinguished by any of the measures tested. This was the only case. These records, as can be seen in Figure 2, are very dichotomic, and it is very likely that just a few ordinal patterns monopolise most of the matches, making it difficult to correctly capture the differences between records [26]. In other words, it is not only the number of patterns found, as with such a regular time series, the motif histogram will be extremely biased.

The results for WPE also confirmed that the number of actual patterns found carries significant discriminant information. This method has already demonstrated its performance in previous studies [12,15], and the replacement of the weights as the normalisation factor by the number of different patterns found, as for PE (WPE + patterns in Table 5), further improved the WPE performance in most of the experiments. This fact suggests that the PE2 approach can be a transversal improvement that could be applied to many methods at the normalisation stage in order to enhance their discriminating power, not just as a method on its own.

A case that deserved further investigation was the PAF dataset, since this was the only case with better PE1 performance, despite significant differences and low variability in number of patterns between the two classes (Table 4). The hypothesis was that, somehow, the information provided by ordinal patterns and number of patterns cancelled each other out. A straightforward approach to solve this problem was to change the arithmetic operation in which *T* was involved, use a multiplication instead of a division. The PAF experiments were repeated with this PE2 algorithm tweak, whose results are shown in Table 8. PE2 performance improved significantly and again was better than PE1.

The results in Table 8 confirm that the patterns found provide discriminant information, but maybe in some specific cases the approach should be slightly different in terms of integration with ordinal patterns. This experiment was repeated for some records that already exhibited good performances using the initial approach described in Algorithm 1. These additional results, shown in Table 9, confirm PE2 is a robust approach in general, and only a few specific cases, such as that of PAF, need a more customised scheme. However, even with both approaches, it was impossible to find significant differences for the HOUSE database.

Although the desnormalisation can be detrimental for low *m* values when not all the records have the same length, for greater *m* values the forbidden patterns become more apparent and the additional information provided should make PE2 outperform PE1. In other words, in the worst case PE2 and PE1 should be equivalent in terms of classification accuracy, but for real time series, which usually exhibit some degree of determinism, PE2 will yield the best performance. With the two approaches proposed, PE2 is a clear winner over PE1.

Despite the $p⇐p_{i}\leftarrow c_{i}*T$ change applied to the PAF case, the new method proposed is still that described initially in Algorithm 1. This final adjustment hints that there might not be a single way to integrate the information of the relative frequency of motifs with its absolute number, and other studies will be necessary to find a better combination and a more optimised algorithm. We tried to keep a very similar algorithm to PE to facilitate adoption and implementation, but there is room for PE2 improvement with regard to generalisation, performance, and even normalisation that will have to be addressed in further studies.

The results for the GAIT2 database were not significant (all GAIT records cut short to 200 samples), and therefore, were far worse than those for GAIT. It could be argued that differences in the GAIT case were mainly due to differences in lengths, and this could be the case when comparing old–healthy with old–Parkinson’s, and young–healthy with old–Parkinson’s, since the lengths were 800–200 originally in both cases. However, the comparison of old–healthy with young–healthy became not possible as well, and this pair had the same length in the GAIT database. Therefore, it can be arguably stated that the lack of significance was due to the insufficient length, since 200 samples are borderline [26], not the lack of differences in length, another factor that requires further research. Anyway, the important point is the comparative analysis between PE1 and PE2.

It could also be hypothesised that the differences in number of patterns found would suffice to classify the records. In order to assess this point, a few experiments were repeated using the complete PE2 method and only the number of actual patterns found as single classification features. Although in some cases the performances of both approaches were very similar (for example, for the BONN database, with m=6, the performances were PE2 = 0.935 vs. 0.930), in others, they were quite different (For the RATS dataset, with m=6, the performances were PE2 = 0.78 vs. 0.72). Since the computational cost of both approaches is almost the same (the algorithm should be run almost completely; only the final Shannon calculation could be omitted), such a simplification is not advisable because not all the differences only lie on the number of admissible patterns.

The parameter and noise influence analysis provided an additional insight into the PE2 approach capabilities. The comparative length influence analysis between PE1 and PE2 in Table 6 showed that both metrics exhibit a similar robustness against *N* variations and short lengths. For extremely short lengths, 50 samples, it was not possible to find differences between classes, which is understandable since 50 samples do not suffice to provide a reasonable pattern frequency estimation. At 250 samples, most of the results became significant, but it was at 500 samples where both methods provided significant classifications in all cases, except PE1 for the WORMS database. With 750 samples, performances were really close to those achieved with the entire records.

The performances of PE1 and PE2 with regard to noise interference seemed very similar, except for the PAF dataset (Table 7). This could be due to the fact that noise impact can also be considered in terms of new ordinal patterns; random time series usually contain all the possible m! patterns as a sign of non-determinism. Therefore, since the difference between PE1 and PE2 lies on the number of patterns found, it can arguably be assumed that, in general, as the noise level increases, PE2 will converge to the PE1 performance, since the differences in terms of number of patterns will become blurred.

PE2 is derived from PE1, and without specific studies yet, it would be sensitive to assume that some PE1 drawbacks can be inherited by PE2. Thus, PE1 could be influenced by amplitude differences in the ordinal patterns [15], by ties [17], or by the record length [26]. A similar set of characterisation studies would be necessary specifically for PE2 to shed some light on these possible issues.

## 5. Conclusions

Every year quite a number of new tools to quantify the dynamical features of time series are described in the scientific literature. These new tools are claimed to be more efficient in algorithmic terms, more sensitive, more robust, or less dependent on input parameters, among many other possible benefits.

As such, PE2 was introduced as an improvement over PE by taking advantage of the differences between time series classes in terms of numbers of different patterns actually found. The present study assessed this discriminant power using several real-life datasets, and we could conclude that the discriminating capabilities of ordinal patterns’ relative frequencies and their counts are clearly complementary and synergistic. This led us to try to combine both measures in a single method to take advantage of their strengths and simultaneously minimise their possible weaknesses. The scheme used for PE using dynamic lists provided the algorithmic template to merge ordinal pattern and pattern number information together by changing the way histogram bins were normalised, and keeping the algorithm’s implementation simple and similar to its ancestor PE.

According to the results obtained, the PE2 approach can be considered a very promising tool in the field of symbolic dynamics. It should not be claimed to be a *cure-all* method, but the classification performance confirmed the hypothesis, and PE2 seems to be able to seamlessly exploit the synergy between PE and the number of patterns found in most cases. It was clearly more robust, since statistical significance was reached in six out of the seven datasets, two more than with PE1 (PE). It also achieved the maximum performance of the two methods tested in five cases, or six if the final PE2 algorithm tweaking is considered (Section 4).

The PE2 algorithm is just a little bit more complex than that of PE, but more memory efficient, since it only needs to store the patterns found, not all the possible m! ones. Moreover, the algorithm introduced can be easily further optimised. In addition to implementation issues to save memory requirements or computational cost, the algorithm could be improved in terms of ordinal and pattern number influences on the final calculations by using a normalisation or weighting scheme based on an additional parameter, such as A/1−A, as in [14]. Additionally, other factors could be included as additional symbols in the motifs. This way, other properties such as sequence amplitude, would become part of the comparisons. In fact, we included the *q* parameter in the PE2 method, as described in [13], and the classification performance of PE2 increased in some cases. The experiments with WPE and WPE+Patterns also confirmed this point.

This new approach will need further studies using other databases and other integration schemes. The influences of equal values in the subsequences [17], and time delay τ, could also be characterised. Further integration with other PE improvements could be worth exploring [6,12,13,14,16] in future.

## Figures and Tables

**Table 1:** Performances achieved using the synthetic records, including the number of patterns found in each case.

Method		m=3	4	5	6	7	8
PE1	Accuracy	1±0	0.65±0.04	0.86±0.07	0.97±0.01	0.66±0.09	0.71±0.08
PE2	Accuracy	0.83±0.01	0.87±0.09	0.91±0.03	1±0	1±0	0.99±0.01
Patterns	Class 1	4.32±0.46	8.30±0.45	19.51±0.63	40.85±1.63	93.14±2.99	182.82±5.79
found	Class 2	4.33±0.47	10.01±0.76	22.71±1.50	53.35±1.91	117.08±5.79	209.71±8.65

**Table 2:** Classification results achieved using PE1 applied to the real experimental dataset. Classification performance is quantified with three parameters: sensitivity, specificity, and accuracy.

	m=3	4	5	6	7	8
BONN	0.93, 0.90, 0.91	0.93, 0.89, 0.91	0.92, 0.89, 0.90	0.91, 0.89, 0.90	0.93, 0.85, 0.89	0.90, 0.85, 0.87
	p<0.0001	p<0.0001	p<0.0001	p<0.0001	p<0.0001	p<0.0001
GAIT	0.40, 1, 0.70	0.60, 1, 0.80	1, 1, 1	1, 1, 1	1, 1, 1	1, 1, 1
	p=0.9168	p=0.2505	p=0.0090	p=0.0090	p=0.0090	p=0.0090
	0.60, 0.80, 0.70	0.40, 1, 0.70	0.80, 0.80, 0.80	1, 0.80, 0.90	1, 0.80, 0.90	1, 0.60, 0.80
	p=0.4647	p=0.4647	p=0.1171	p=0.0282	p=0.0282	p=0.1171
	0.80, 0.60, 0.70	0.60, 0.80, 0.70	1, 1, 1	1, 1, 1	1, 1, 1	1, 1, 1
	p=0.6015	p=0.6015	p=0.0090	p=0.0090	p=0.0090	p=0.0090
GAIT2	0.60, 0.80, 0.70	0.80, 0.80, 0.80	0.80, 0.60, 0.70	1, 0.60, 0.80	1, 0.40, 0.70	0.60, 0.60, 0.60
	p=0.4647	p=0.0758	p=0.7540	p=0.4647	p=0.7540	p=0.7540
	0.60, 0.60, 0.60	1, 0.60, 0.80	0.80, 0.80, 0.80	1, 0.60, 0.80	0.80, 0.40, 0.60	0.40, 0.80, 0.60
	p=0.9168	p=0.2505	p=0.2505	p=0.3472	p=0.8335	p=1
	0.80, 0.60, 0.70	1, 0.40, 0.70	0.60, 0.60, 0.60	0.60, 0.60, 0.60	0.60, 0.60, 0.60	0.60, 0.60, 0.60
	p=0.4647	p=0.6015	p=0.9168	p=0.9168	p=0.5970	0.9157
FANT	0.66, 0.66, 0.66	0.88, 0.55, 0.72	0.77, 0.66, 0.72	0.77, 0.66, 0.72	0.77, 0.66, 0.72	0.66, 0.77, 0.72
	p=0.4015	p=0.2696	p=0.1451	p=0.1451	p=0.1222	p=0.1023
RATS	0.75, 0.55, 0.65	0.7, 0.65, 0.675	0.55, 0.65, 0.60	0.75, 0.50, 0.625	0.70, 0.50, 0.60	0.45, 0.75, 0.60
	p=0.2792	p=0.2133	p=0.2674	p=0.4488	p=0.7867	p=0.4651
WORMS	0.61, 0.67, 0.65	0.60, 0.71, 0.66	0.65, 0.68, 0.67	0.64, 0.70, 0.67	0.65, 0.70, 0.68	0.65, 0.70, 0.68
	p=0.0405	p=0.0402	p=0.0340	p=0.0271	p=0.0177	p=0.0096
HOUSE	0.70, 0.60, 0.65	0.75, 0.55, 0.65	0.65, 0.60, 0.62	0.70, 0.60, 0.65	0.75, 0.50, 0.62	0.70, 0.55, 0.62
	p=0.1941	p=0.1762	p=0.1941	p=0.1440	p=0.1677	p=0.1198
PAF	0.76, 0.88, 0.82	0.80, 0.84, 0.82	0.80, 0.80, 0.80	0.92, 0.72, 0.82	0.96, 0.68, 0.82	0.92, 0.68, 0.80
	p<0.0001	p<0.0001	p<0.0001	p<0.0001	p<0.0001	p=0.0002

**Table 3:** Classification results achieved using the new approach, PE2, applied to all the time series databases. Classification performance is quantified with three parameters: sensitivity, specificity, and accuracy.

Dataset	m=3	4	5	6	7	8
BONN	0.93, 0.89 ,0.91	0.93, 0.89, 0.91	0.90, 0.97, 0.93	0.91, 0.90, 0.90	0.46, 0.79, 0.62	0.91, 0.81, 0.86
	p<0.0001	p<0.0001	p<0.0001	p<0.0001	p=0.1782	p<0.0001
GAIT	1, 1, 1	1, 1, 1	1, 1, 1	1, 1, 1	1, 1, 1	1, 1, 1
	p=0.0090	p=0.0090	p=0.0090	p=0.0090	p=0.0090	p=0.0090
	0.60, 0.80, 0.70	0.60, 0.80, 0.70	1, 0.80, 0.90	0.60, 0.80, 0.70	1, 0.40, 0.70	0.80, 0.80, 0.80
	p=0.2505	p=0.2505	p=0.0472	p=0.9168	p=0.9168	p=0.2505
	1, 1, 1	1, 1, 1	1, 1, 1	1, 1, 1	1, 1, 1	1, 1, 1
	p=0.0090	p=0.0090	p=0.0090	p=0.0090	p=0.0090	p=0.0090
GAIT2	0.60, 0.80, 0.70	0.80, 0.80, 0.80	0.80, 0.60, 0.70	0.60, 1, 0.80	0.40, 1, 0.70	0.60, 0.60, 0.60
	p=0.4647	p=0.0758	p=0.6015	p=0.6015	p=0.6015	p=0.7373
	0.60, 0.60, 0.60	1, 0.60, 0.80	0.60, 0.60, 0.60	0.60, 1, 0.80	0.40, 0.80, 0.70	0.60, 0.60, 0.60
	p=0.9168	p=0.2505	p=0.9168	p=0.4647	p=1	p=1
	0.80, 0.60, 0.70	1, 0.40, 0.70	0.60, 0.80, 0.70	0.60, 0.60, 0.60	0.60, 0.60, 0.60	0.40, 0.60, 0.50
	p=0.4647	p=0.6015	p=0.7540	p=0.9168	p=0.5970	p=0.9112
FANT	0.65, 0.60, 0.62	0.9, 0.50, 0.70	0.70, 0.60, 0.65	0.85, 0.50, 0.67	0.70, 0.80, 0.75	0.45, 0.75, 0.60
	p=0.0514	p=0.0619	p=0.0883	p=0.0483	p=0.0024	p=0.1595
RATS	0.66, 0.66, 0.66	0.77, 0.77, 0.77	0.77, 0.77, 0.77	0.77, 0.77, 0.77	0.77, 0.77, 0.77	0.77, 1, 0.88
	p=0.4015	p=0.0469	p=0.0379	p=0.0379	p=0.151	p=0.0091
WORMS	0.61, 0.67, 0.65	0.60, 0.71, 0.66	0.65, 0.68, 0.67	0.73, 0.68, 0.70	0.65, 0.52, 0.58	0.55, 0.51, 0.52
	p=0.0405	p=0.0511	p=0.0386	p<0.0001	p=0.0874	p=0.6569
HOUSE	0.70, 0.60, 0.65	0.75, 0.55, 0.65	0.75, 0.50, 0.62	0.55, 0.70, 0.62	0.60, 0.65, 0.62	0.55, 0.70, 0.62
	p=0.1941	p=0.1762	p=0.2339	p=0.1677	p=0.2335	p=0.1231
PAF	0.60, 0.76, 0.68	0.60, 0.48, 0.54	0.80, 0.68, 0.74	0.60, 0.56, 0.58	0.72, 0.52, 0.62	0.56, 0.60, 0.58
	p=0.0141	p=0.7341	p=0.0064	p=0.2815	p=0.3669	p=0.2815

**Table 4:** Actual number of patterns found in each dataset in terms of mean value ± standard deviation.

	m=3	4	5	6	7	8
BONN	6±0/6	24±0/24	113.10±5.37/120	374±57.17/720	780.43±154.06/5040	1288.65±269.85/40,320
	6±0/6	23.03±1.54/24	79.87±17.00/120	192.42±61.17/720	369.93±127.66/5040	624.79±211.50/40,320
GAIT	6±0/6	24±0/24	100.60±5.88/120	199±25.17/720	230±29.27/5040	235.60±30.18/40,320
	6±0/6	24±0/24	119.60±0.48/120	466.80±15.52/720	734.40±41.62/5040	803.20±57.72/40,320
	6±0/6	24±0/24	119.20±0.74/120	429.80±22.43/720	669.60±45.23/5040	739.20±43.06/40,320
FANT	6±0/6	24±0/24	119.80±0.50/120	616.35±40.47/720	2259.95±282.22/5040	4470.40±572.96/40,320
	6±0/6	24±0/24	119.65±1.10/120	649.65±59.37/720	2428.60±486.31/5040	4592.40±914.47/40,320
RATS	6±0/6	15.77±2.89/24	31.55±10.41/120	54.44±21.48/720	86.78±38.89/5040	132±68.39/40,320
	6±0/6	13.22±1.81/24	25±7.33/120	42.22±16.55/720	65.44±29.84/5040	95.11±46.98/40,320
WORMS	5.93±0.56/6	23.59±2.64/24	96.77±21.11/120	251.07±114.37/720	378.43±188.75/5040	456.52±208.39/40,320
	5.95±0.48/6	23.45±2.38/24	99.80±24.40/120	273.28±108.67/720	425.19±117.21/5040	524.13±203.89/40,320
HOUSE	6±0/6	24±0/24	106.15±14.75/120	358.30±94.82/720	664.90±155.76/5040	854.30±121.47/40,320
	6±0/6	23.95±0.21/24	110.40±11.40/120	396.90±92.47/720	722.90±142.14/5040	899.45±101.64/40,320
PAF	6±0/6	24±0/24	108.44±12.88/120	300.48±68.53/720	406.92±94.72/5040	437.52±92.95/40,320
	6±0/6	23.52±0.94/24	87.24±15.42/120	199.48±45.72/720	286.88±51.14/5040	335.88±46.67/40,320

**Table 5:** Summary of the performances achieved by PE1 and PE2, including comparison with the same approach applied to WPE. Only the best significant case is reported, including the corresponding *m* value, in terms of classification accuracy only. If statistical significance was not achieved, the performance was labelled NS.

Dataset	PE1	PE2	WPE	WPE+Patterns
BONN	m=3	m=5	m=3	m=3
	0.91	0.93	0.80	0.96
GAIT	m=7	m=5	m=5	m=4
	1	1	0.90	0.90
	0.90	0.90	NS	0.80
	1	1	0.90	1
FANT	NS	m=7	m=8	m=6
		0.75	0.82	0.77
RATS	NS	m=8	m=3	m=3
		0.88	0.77	0.94
WORMS	m=8	m=6	m=6	m=6
	0.68	0.70	NS	NS
HOUSE	NS	NS	m=8	m=8
			0.72	0.90
PAF	m=6	m=5	m=4	m=4
	0.82	0.74	0.80	0.86

**Table 6:** Accuracy achieved for different lengths *N*. Values tested were 50, 250, 500, and 750. Datasets had to be at least 750 samples long. Embedded dimension was chosen in order to get the most similar performances for PE1 and PE2, provided the classification had statistical significance.

Dataset	Method	N=50	250	500	750
BONN (m=6)	PE1	0.67	0.78	0.83	0.83
	PE2	0.62	0.65	0.73	0.74
GAIT (m=5)	PE1	(0.60, 0.60, 0.70)	(0.70, 0.70, 0.70)	(1, 0.70, 1)	(1, 0.70, 1)
	PE2	(0.50, 0.60, 0.70)	(0.80, 0.70, 0.70)	(1, 0.70, 1)	(1, 0.80, 1)
WORMS (m=5)	PE1	0.61	0.61	0.63	0.65
	PE2	0.59	0.62	0.68	0.71
Synthetic (m=6)	PE1	0.56±0.01	0.77±0.03	0.92±0.04	0.96±0.02
	PE2	0.60±0.01	0.90±0.01	0.91±0.02	0.99±0.01

**Table 7:** Accuracy achieved for different levels of synthetic noise. Values tested were 40, 30, 25, 20, and 15dB SNR. Embedded dimension was chosen in order to get the most similar performances of PE1 and PE2, provided the classification had statistical significance. GAIT results only correspond to a single case (between healthy young and old adults), since the others were 1 for all the tests.

Dataset	Method	SNR=40 dB	30 dB	25 dB	20 dB	15 dB
BONN (m=6)	PE1	0.87±0.00	0.81±0.00	0.78±0.01	0.78±0.01	0.80±0.00
	PE2	0.87±0.00	0.78±0.00	0.78±0.01	0.83±0.01	0.83±0.01
GAIT (m=5)	PE1	0.80±0.10	0.63±0.05	0.80±0.10	0.66±0.11	0.63±0.05
	PE2	0.80±0.00	0.76±0.05	0.80±0.00	0.73±0.05	0.70±0.00
WORMS (m=5)	PE1	0.66±0.01	0.65±0.01	NS	NS	NS
	PE2	0.66±0.01	0.65±0.01	NS	NS	NS
PAF (m=5)	PE1	NS	NS	NS	NS	NS
	PE2	0.72±0.00	0.70±0.00	0.70±0.02	0.70±0.01	0.71±0.01
Synthetic (m=6)	PE1	1±0.00	0.85±0.22	0.89±0.01	0.78±0.03	NS
	PE2	0.99±0.00	0.83±0.23	0.83±0.02	0.74±0.06	NS

**Table 8:** Classification results achieved using the new approach, PE2, applied to the PAF dataset, and $p⇐p_{i}\leftarrow c_{i}*T$ instead of $p⇐p_{i}\leftarrow \frac{c_{i}}{T}$.

Dataset	m=3	4	5	6	7	8
PAF	0.68, 0.72, 0.70	0.64, 0.76, 0.70	0.72, 0.96, 0.84	0.72, 0.88, 0.80	0.72, 0.92, 0.82	0.68, 0.84, 0.76
	p=0.0080	p=0.0067	p<0.0001	p<0.0001	p=0.0003	p=0.0009

**Table 9:** Classification results achieved using the new approach PE2, applied to other datasets, and $p⇐p_{i}\leftarrow c_{i}*T$ instead of $p⇐p_{i}\leftarrow \frac{c_{i}}{T}$.

Dataset	m=3	4	5	6	7	8
BONN	0.93, 0.90, 0.91	0.49, 0.84, 0.66	0.92, 0.92, 0.92	0.93, 0.93, 0.93	0.92, 0.94, 0.93	0.92, 0.94, 0.93
	p<0.0001	p=0.0137	p<0.0001	p<0.0001	p<0.0001	p<0.0001
FANT	0.65, 0.60, 0.62	0.55, 0.75, 0.65	0.75, 0.60, 0.67	0.80, 0.45, 0.62	0.60, 0.55, 0.57	0.85, 0.45, 0.65
	p=0.0547	p=0.0547	p=0.0398	p=0.4488	p=0.7660	p=0.1440
WORMS	0.60, 0.68, 0.65	0.63, 0.70, 0.67	0.68, 0.51, 0.58	0.67, 0.59, 0.62	0.68, 0.64, 0.66	0.70, 0.65, 0.67
	p=0.0494	p=0.0015	p=0.1698	p=0.1301	p=0.0564	p=0.0300
